# Assessment of p.Phe508del-CFTR functional restoration in pediatric primary cystic fibrosis airway epithelial cells

**DOI:** 10.1371/journal.pone.0191618

**Published:** 2018-01-23

**Authors:** Erika N. Sutanto, Amelia Scaffidi, Luke W. Garratt, Kevin Looi, Clara J. Foo, Michela A. Tessari, Richard A. Janssen, David F. Fischer, Stephen M. Stick, Anthony Kicic, on behalf of AREST CF

**Affiliations:** 1 Telethon Kids Institute, the University of Western Australia, Nedlands, Western Australia, Australia; 2 Department of Respiratory Medicine, Princess Margaret Hospital for Children, Perth, Western Australia, Australia; 3 Office of Research Enterprise, The University of Western Australia, Nedlands, Western Australia, Australia; 4 School of Paediatrics and Child Health, The University of Western Australia, Nedlands, Western Australia, Australia; 5 Galapagos BV, 2300 AC, Leiden, the Netherlands; 6 Charles River Laboratories, Leiden, the Netherlands; 7 Centre for Cell Therapy and Regenerative Medicine, School of Medicine and Pharmacology, The University of Western Australia, Nedlands, Western Australia, Australia; 8 Department of Respiratory Medicine, Royal Children’s Hospital, Melbourne, Australia; 9 Murdoch Children’s Research Institute, Melbourne, Australia; University of Alabama at Birmingham, UNITED STATES

## Abstract

**Background:**

Mutations in the cystic fibrosis transmembrane regulator (CFTR) gene can reduce function of the CFTR ion channel activity and impair cellular chloride secretion. The gold standard method to assess CFTR function of ion transport using the Ussing chamber requires a high number of airway epithelial cells grown at air-liquid interface, limiting the application of this method for high throughput screening of potential therapeutic compounds in primary airway epithelial cells (pAECs) featuring less common CFTR mutations. This study assessed an alternative approach, using a small scale halide assay that can be adapted for a personalized high throughput setting to analyze CFTR function of pAEC.

**Methods:**

Pediatric pAECs derived from children with CF (pAEC_CF_) were established and expanded as monolayer cultures, before seeding into 96-well plates for the halide assay. Cells were then transduced with an adenoviral construct containing yellow fluorescent protein (eYFP) reporter gene, alone or in combination with either wild-type CFTR (WT-CFTR) or p.Phe508del CFTR. Four days post transduction, cells were stimulated with forskolin and genistein, and assessed for quenching of the eYFP signal following injection of iodide solution into the assay media.

**Results:**

Data showed that pAEC_CF_ can express eYFP at high efficiency following transduction with the eYFP construct. The halide assay was able to discriminate functional restoration of CFTR in pAEC_CF_ treated with either WT-CFTR construct or the positive controls syntaxin 8 and B-cell receptor-associated protein 31 shRNAs.

**Significance:**

The current study demonstrates that the halide assay can be adapted for pediatric pAEC_CF_ to evaluate restoration of CFTR function. With the ongoing development of small molecules to modulate the folding and/or activity of various mutated CFTR proteins, this halide assay presents a small-scale personalized screening platform that could assess therapeutic potential of molecules across a broad range of CFTR mutations.

## Introduction

Cystic fibrosis (CF) manifests as a multi-organ disease, however, lung disease presenting with recurrent infections, chronic neutrophilic inflammation and structural pathologies remain the primary cause of mortality [1,2]. The molecular basis of CF is a functionally defective ion channel, caused by inheritable mutations in the cystic fibrosis transmembrane conductance regulator (CFTR) gene. Though more than 1000 mutations have been identified [1], the most common mutation is a codon deletion in exon 10 for phenylalanine at position 508 (p.Phe508del) in the encoded CFTR polypeptide [3]. This leads to defective trafficking of the mutant protein to the cell membrane and also compromises the transport of chloride ions [4]. More than 90% of patients with CF have at least one p.Phe508del allele [5] and individuals bearing this mutation are often associated with more severe phenotype [3].

In the lung, the CFTR protein is highly expressed at the apical surface of epithelial cells in the airways [6] and its primary function is to help regulate the airway liquid microenvironment through secretion of chloride ions and other molecules. However, defective function in CF airways leads to a significantly altered airway environment, characterized by insufficient mucociliary clearance that is further complicated by the secondary effects of recurrent, destructive infections [2]. Airway epithelial cells (AECs) have been identified as highly relevant targets for correction of CFTR function. However, development of potential therapeutics relies on functional assays to quantify their effect on CFTR. The current gold standard method of using Ussing chamber to measure ion transport through electrophysiology requires a high number of AECs for each permeable insert grown at air-liquid interface (ALI), which precludes the use of primary AEC (pAEC) from pediatric CF populations. With the discovery that small molecules can have the potential to actively correct CFTR and many more that are currently in the pipeline especially for rare mutations of CFTR, a small scale high-throughput screening (HTS) platform is necessary to help realize personalized medicine approaches in early CF. One such approach would be to adapt a halide sensitive fluorescent reporter molecule for expression in pAEC and its utilization in an assay that assesses ion channel activity.

Verkman and colleagues [7] first reported measuring chloride concentrations via fluorescent indicators based upon heterocyclic organic compounds with quaternary nitrogen like quinolinium. Follow-up studies investigated quinolinium salt-based halide sensitive fluorescent probes such as (6-methoxy-N-9-sulphopropyl)quinolinium (SPQ) and N-(ethoxycarbonylmethyl)-6-methoxyquinolinium bromide (MQAE) [8,9], before green fluorescent protein (GFP) was modified into a halide-sensitive indicator that measure chloride transport in epithelial cells [10,11].

Many studies have assessed CFTR restoration using transformed cell lines and animal models [12–14], however, applying these investigations to freshly isolated primary cell populations that better reflect natural biological variety poses some new and unique challenges. There have only been three reports examining correction of p.Phe508del expression and function in pAEC cultures [15–17] and all were conducted using adult-derived cells. Since pediatric populations present the ideal opportunity for early intervention, the aim of the present study was to investigate whether functional restoration of p.Phe508del CFTR could be efficiently assessed in pediatric-derived cystic fibrosis airway epithelial cells (pAEC_CF_) using an adapted adenoviral-based halide assay.

## Materials and methods

### Reagents

Bronchial Epithelium Basal Media (BEBM®) was purchased from Lonza (Clonetics®, Walkersville, MD, USA); bovine pituitary extract, bovine serum albumin, fibronectin, hydrocortisone, recombinant human epidermal growth factor, epinephrine hydrochloride, transferrin, triiodothyronine, insulin, and retinoic acid were obtained from Sigma-Aldrich Pty. Ltd. (St. Louis, MO, USA); trypsin/EDTA, penicillin/streptomycin, gentamycin, and fungizone from Life Technologies Pty. Ltd (Melbourne, Australia); bovine collagen Type I from Becton Dickinson Pty. Ltd (Bedford, MA, USA); synthetic serum Ultroser G acquired from Ciphergen (Cergy-Saint-Christophe, France).

### Subjects

For this study, 16 children with CF (7 male and 9 female children)under 7 years of age were recruited when they came to hospital for their annual early surveillance visit [18]. This study was approved by the Human Ethics Committee of Princess Margaret Hospital (1762/EP) and written consent was obtained from the parents or legal guardians of each participant after being fully informed about the nature and purpose of this study. Detailed characteristics of donor subjects are provided in Table 1.

**Table 1:** Demographics and characteristic of the study population at the time of cell sampling.

ID	Sex	Age (years)	Genotype	Pulmonary infection (cfu/ml)	IL-8 (pg/ml)	Neutrophils (x10^3^/ml)
1	M	4.1	p.Gly551Asp/p.Arg117His	Mixed oral flora (10^7^ cfu/ml)	220	43.71
2	M	0.2	p.Phe508del/p.Phe508del	None	620	18.46
3	M	5.2	p.Phe508del/p.Phe508del	None	1690	90.46
4	F	3.2	p.Phe508del/p.Phe508del	None	50	6.65
5	F	1.3	p.Phe508del/p.Phe508del	None	240	8.19
6	F	0.3	p.Phe508del/p.Phe508del	None	290	39.60
7	F	6.2	p.Phe508del/p.Phe508del	*S*. *Aureus* (10^5^ cfu/ml), mixed oral flora (10^7^ cfu/ml)	1060	37.10
8	F	5.2	Phe508del/unknown	Mixed oral flora (10^6^ cfu/ml)	440	64.91
9	F	3.0	p.Phe508del/p.Phe508del	Mixed oral flora (10^3^ cfu/ml)	2380	197.86
10	M	5.3	p.Phe508del/p.Phe508del	Mixed oral flora (10^3^ cfu/ml)	290	5.58
11	F	1.9	p.Phe508del/p.Phe508del	N/A	800	91.61
12	M	5.1	p.Phe508del/p.Phe508del	N/A	1170	21.1
13	F	5.1	p.Phe508del/p.Phe508del	N/A	650	162.9
14	M	4.1	p.Phe508del/p.Gly551Asp	N/A	1800	N/A
15	M	0.3	c.2052delA/p.Ser549Asn	N/A	800	10.26
16	F	0.3	p.Phe508del/p.Arg117His	N/A	100	31.53

### Establishment of pAEC_CF_ cultures

The pAEC were collected via brushing of the tracheal mucosa as previously described [19–21]. Brushes were collected and transported back to the laboratory on ice in media consisting of RPMI-1640 [19,21]. Cells were then detached from the cytology brush tips by vortexing and subsequent centrifugation at 500 *g* for 7 minutes at 4°C. The resulting cell pellet was then resuspended in complete growth medium of BEBM supplemented with bovine pituitary extract (50 μg/ml), hydrocortisone (0.5 μg/ml), epidermal growth factor (0.5 ng/ml), epinephrine (0.5 μg/ml), triiodothyronine (6.5 ng/ml), insulin (5 μg/ml), transferrin (10 μg/ml), fungizone (2.5 μg/ml), penicillin/streptomycin (100 Units/ml penicillin and 0.1 mg/ml streptomycin) and gentamycin (50 μg/ml). Macrophages were removed via incubating cell solution on a dish coated with anti-CD68 antibody. Following cell count, pAEC were seeded for culture establishment into new flasks pre-coated with a solution of 10mM fibronectin, 30mM collagen and 100mM bovine serum albumin. Epithelial lineage of established pAEC cultures has been previously confirmed [19] with positive immunocytochemical staining for cytokeratin 19 (CK19; epithelial marker) and negative staining for vimentin (mesenchymal marker), CD1a (dendritic) and von Willebrand factor (endothelial marker).

### pAEC_CF_ culture maintenance

Established submerged monolayer cultures were maintained by feeding every second day with fresh complete growth media and once confluent, cells were passaged by incubating them in 0.25% Trypsin/0.05% EDTA for 7 minutes at 37°C. Cells were pelleted via centrifugation (500*g* for 7 minutes at 4°C) and were subsequently seeded in new culture flasks in complete growth media. Cultures were further expanded as necessary once reaching 100% confluence. Following expansion from the initial culture, cells were then utilized for halide assay at passage 2. Here, cells were plated at density of 20,000 cells per well in 96-well black microplates (Greiner Bio-One, Frickenhausen, Germany) and incubated for an additional 48 hours in complete growth media prior to commencing the assay. Cells were seeded typically in triplicate wells for each of the parameters tested.

### Adenoviral vectors

To efficiently express a halide-sensitive enhanced yellow fluorescent protein (eYFP) in pAEC, cDNA was expressed from adenovirus deleted for adenoviral genes E1 and E2A with adenoviral type Ad5. The cDNA for p.Phe508del CFTR and wild type CFTR (WT-CFTR), as well as short hairpin RNAs (shRNAs) that inhibited syntaxin 8 (STX8) or B-cell receptor-associated protein 31 (BCAP-31) RNA, were also cloned in the same adenoviral vector type Ad5. Adenoviral titres were determined by quantitative PCR and defined as adenoviral particles (VP) accordingly.

### pAEC_CF_ transduction efficiency and cytotoxicity

In order to ensure that our pAEC_CF_ could be efficiently transduced by adenoviral vectors, cells cultured in 96-well black microplates to 90% confluence were transduced with the eYFP vector at different multiplicities of infection (MOI) of 500, 1000 and 2000 viral particles per cell. Following incubation over 48–96 hours at 37°C in a humidified atmosphere, eYFP fluorescence was imaged via fluorescence microscopy using 488 nm excitation and 525 nm emission wavelengths. The eYFP-expressing cells were then quantified as a percentage of the total number of cells from 10 randomly chosen fields (containing > 100 cells) per sample. Positive cells were then averaged over these 10 fields and then further averaged over triplicate images. Cell cytotoxicity was determined by measuring the level of lactate dehydrogenase (LDH) using the Cytotox 96® Non-radioactive cytotoxicity assay (Promega Corporation, Madison, WI, USA) and performed as per manufacturer’s instructions. Cell viability following transduction was also measured using the CellTiter 96® AQ_ueous_ One Solution Cell Proliferation Assay (Promega) again according to the manufacturer’s instructions.

### Optimization of halide reporter assay

To determine optimal output parameters, pAEC_CF_ were transduced with eYFP at MOI 500 for 96 hours and the fluorescent signal measured on a POLARStar fluorescent plate reader (Optima BMG Lab Technologies, Germany). The plate reader was equipped with 485nm excitation and HQ535/30M emission filters (Chroma Technology Corp., VT, USA), injectors to deliver solution and ability to measure fluorescence from either top or bottom of the microplate.

### CFTR function analysis by halide reporter assay

Functional restoration of CFTR was assessed by the output readings of the halide reporter assay as previously described [7,22]. Briefly, pAEC_CF_ were transduced with eYFP construct alone or in combination with either WT-CFTR or p.Phe508del constructs for 96 hours at 37°C. Cells were then washed in phosphate buffered saline (PBS) and incubated in gluconate buffer containing 50 μM genistein and 10 μM forskolin for 5 minutes at room temperature to stimulate CFTR activity. Next, the eYFP signal was detected using the POLARStar fluorescent plate reader. After the initial 2 seconds reading, 110 μl of iodide solution (PBS modified with 137 mM NaI instead of NaCl) was added to each well. The fluorescence signal was measured for a further 14 seconds with a sampling rate of 200 milliseconds. The resulting fluorescent intensity was then normalized to the readings prior to injection of iodide solution and exponential curve fitted to the data to calculate the rate of iodide flux.

### Statistical analysis

Statistical analysis on data presented in this study was performed using Kruskal-Wallis with Dunn’s multiple comparison test. Statistical significance was indicated by p<0.05.

## Results

### Confirmation of adenovirus transduction efficiency and cytotoxicity in pAEC_CF_

**Fig 1:**
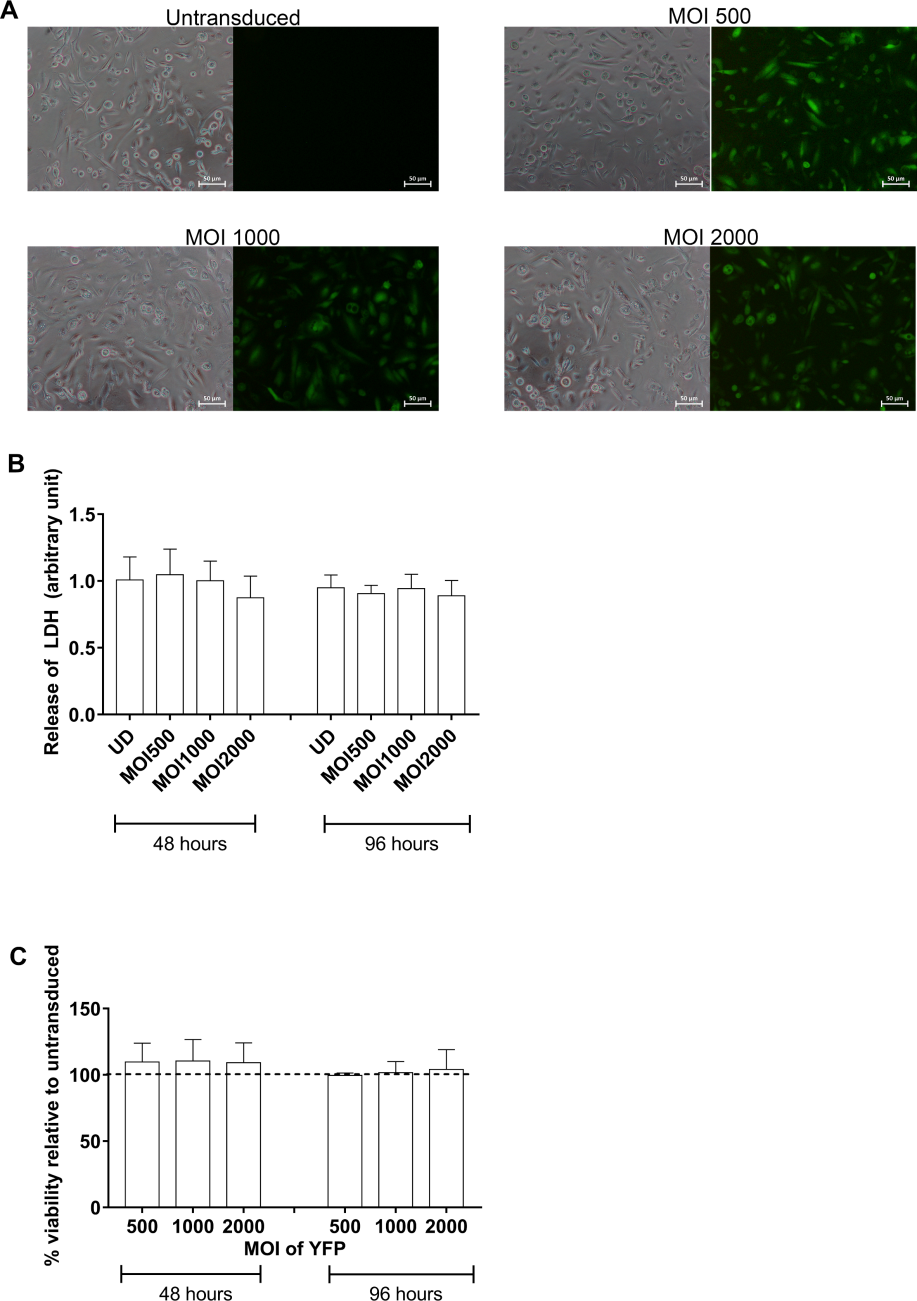
Assessment of transduction efficiency in primary airway epithelial cells from children with CF. Primary AECs from children with CF were seeded into 96-well culture plates, grown to confluence and transduced with adenoviral construct carrying eYFP at a range of MOI (500, 1000 and 2000) over 48 hours. **(A)** Control non-transduced cells exhibited a typical cobblestone morphology and lacked any fluorescence. Following infection at MOI 500, 1000 and 2000, pAEC_CF_ displayed a high transduction efficiency with a majority of cells expressing eYFP. Signal intensity appeared to correlate with increasing MOI with little cytopathic effect. When quantified, more than 85% of cells were found to express eYFP, and that this increased with MOI. (Representative photomicrographs taken using 4x objective magnification from experiments conducted on 3 separate subjects) **(B)** Cell cytotoxicity as measured by LDH production was negligible over all MOI transduction parameters with eYFP and until the completion of the halide assay (n = 4). **(C)** No significant effect on cell viability was observed over the assay period (n = 4). Dash line indicates untransduced cells.

Initial assessment of transduction efficiency by the adenoviral vector in CF primary AEC was required to ascertain that these cells were suitable for assay development. A series of transductions of pAEC_CF_ with different MOI of the adenoviral vector followed by semi-quantitative measurement of eYFP-expressing cells demonstrated that the fluorescent signal intensified with increasing titres of eYFP-encoding adenovirus (Fig 1). Very high transfection efficiency (85.01±1.45%) was achieved at the minimum MOI 500 and there appeared to be no significant cytotoxic effect, as cells retained their typical epithelial morphology. This was confirmed by measuring LDH production and cell viability post transduction which showed no significant difference between untransduced cells and those transduced with different MOI of YFP (Fig 1B and 1C). The expression of eYFP was maintained for the duration of the intended halide assay of 96 hours. Thus, for all subsequent experiments, eYFP at the lowest MOI 500 was used to transduce the pAEC.

### Assessment of transduction by adenoviral constructs in pAEC_CF_

**Fig 2:**
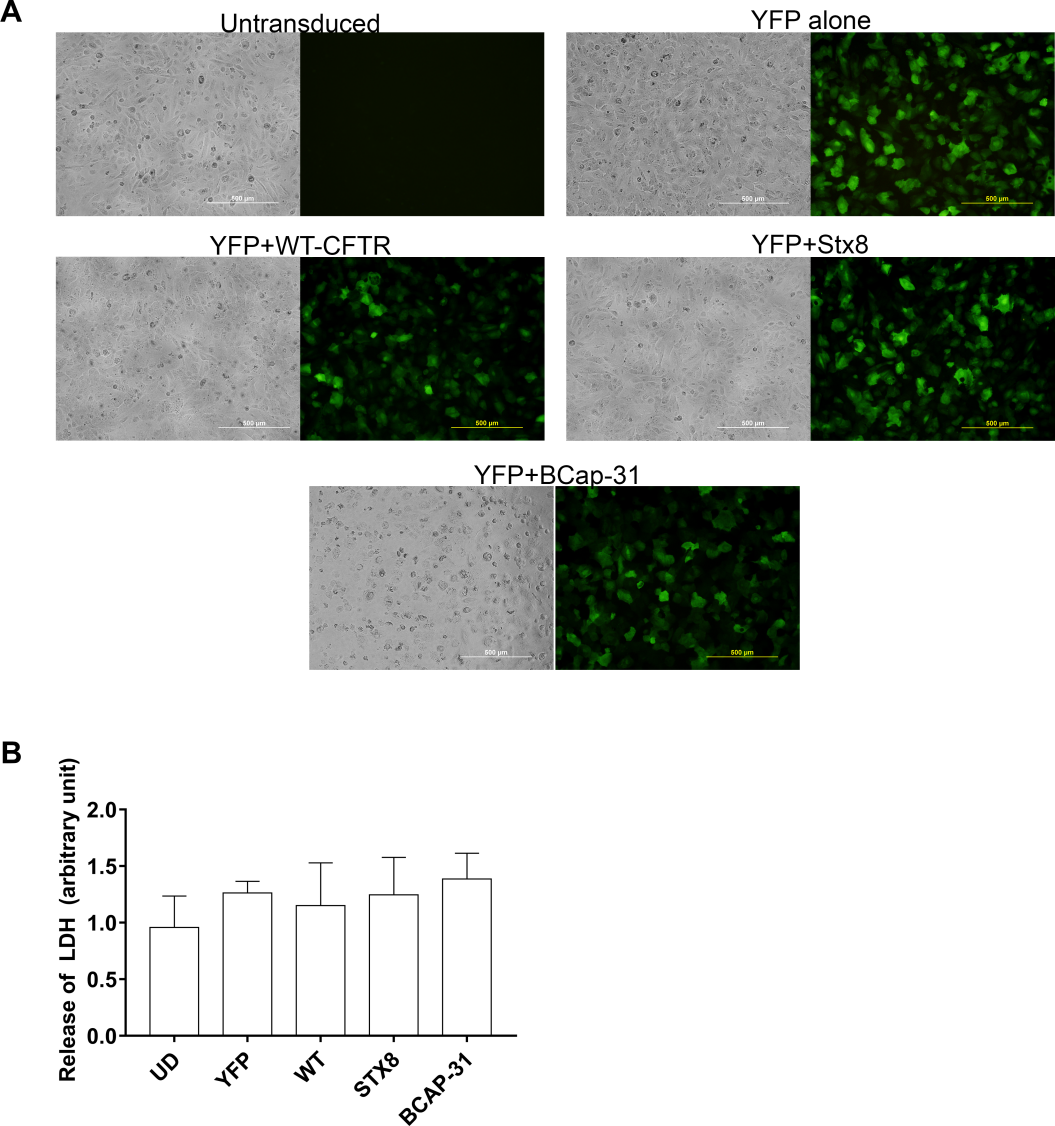
Confirmation of successful transduction of positive controls and WT-CFTR in CF cells. **(A)** Primary AEC from children with CF were seeded into 96-well culture plates and maintained in culture until confluent. Cells were then transduced with adenoviral constructs containing eYFP alone or in combination with wild-type CFTR (WT-CFTR), or BCAP-31 or STX-8 shRNA vectors for 96 hours after which fluorescence micrographs were taken. Results showed minimal auto fluorescence in non-transduced cells, whilst fluorescent signals were detected following transduction with all other constructs. Quantification revealed that greater than 85% of cells expressed eYFP following transduction with any particular construct. (Representative photomicrographs taken using 4x objective magnification from experiments conducted on 4 separate subjects) **(B)** Assessment of LDH release demonstrated minimal cytotoxicity associated with transduction of pAECs with eYFP alone or in combination with WT-CFTR, STX8 or BCAP-31(n = 4).

The next step was to assess transduction of eYFP in combination with transduction by positive shRNA control to determine whether eYFP expression was altered when cells were transduced with more than one construct. Positive controls assessed were WT-CFTR cDNA, as well as shRNA targeting STX8 or BCAP-31, two factors that regulate CFTR trafficking and prevent intracellular migration of defective p.Phe508del CFTR to the plasma membrane [23,24]. Fig 2 represents a series of panels of bright field and fluorescence photomicrographs of pAEC_CF_ in a representative control (non-transduced) sample, eYFP alone, or eYFP in combination with WT-CFTR cDNA, BCAP-31 shRNA or STX8 shRNA. In each setting except the BCAP-31 panel, cells retained their morphology following transduction with minimal cytotoxicity. When transduced with BCAP-31 and eYFP, cells appeared smaller and stressed as indicated by the presence of numerous intracellular vacuoles. Assessment of cell cytotoxicity via LDH release demonstrated no significant cytotoxicity following transduction with eYFP alone or in combination with WT-CFTR, STX8 or BCAP-31. We again quantified the number of eYFP positive cells and found that co-transduction of primary CF AECs with any of the positive control constructs still retained greater than 85% efficiency.

### Determination of optimal output parameters

**Fig 3:**
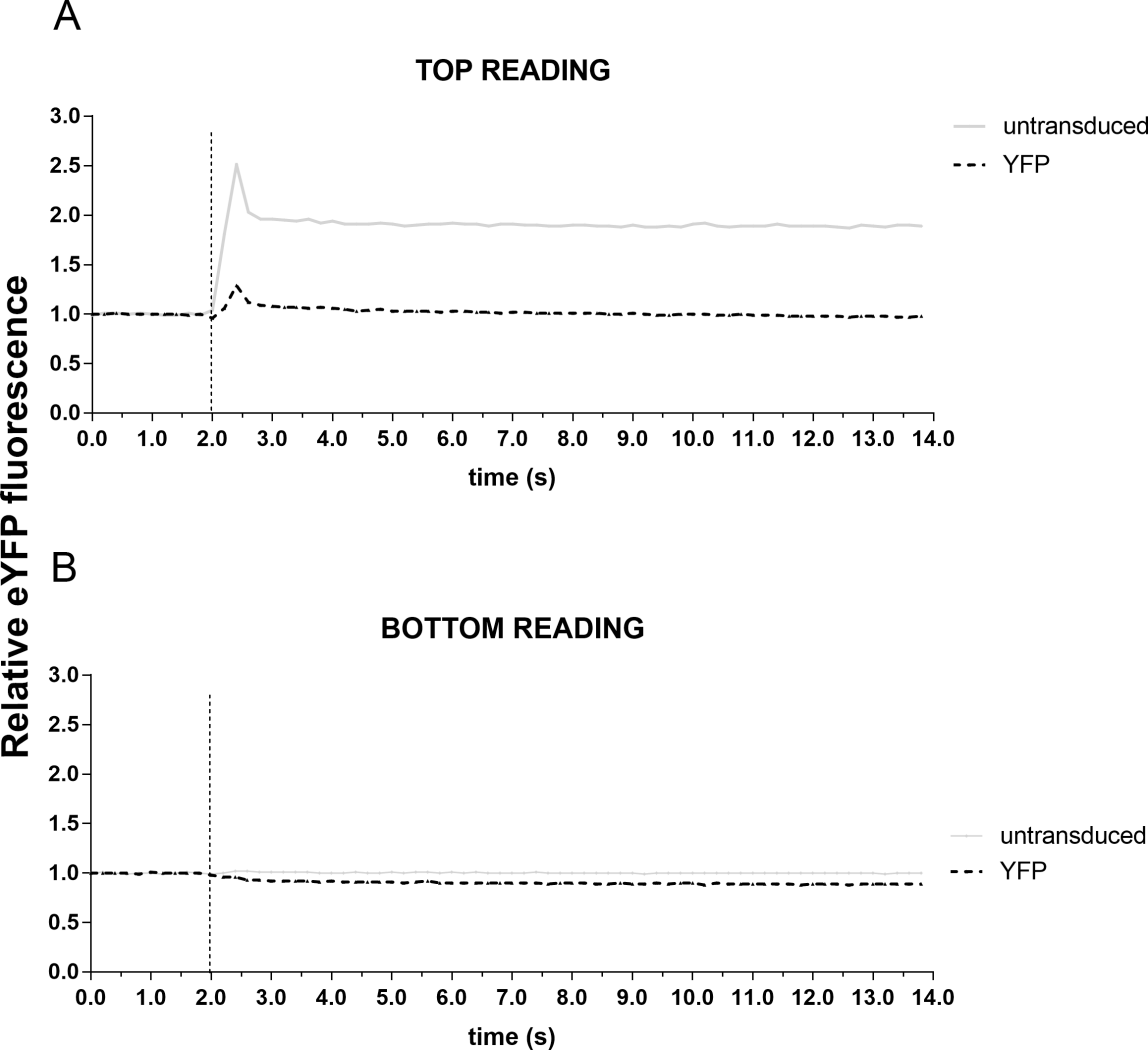
Optimization of output readings from different depths of the culture well. Primary AECs from children with CF were seeded into 96-well culture plates, grown to confluence and transduced with eYFP at MOI 500 for 96 hours. Iodide solution was then injected into each well and eYFP fluorescence then read either from the apical (top) or basal (bottom) location of each well. (**A**) Analysis revealed that top readings resulted in instabilities of fluorescent signal that coincided with the time of iodide injection. Furthermore, there was also inconsistency in the fluorescence signal readouts independent of whether the cells were transduced or not. (**B**) In contrast, bottom reading outputs were more stable and consistent, with no instabilities in fluorescent signal intensity. Therefore, all subsequent halide assay measurements were taken from the bottom of the well.

Having determined that pAEC_CF_ can be successfully and efficiently transduced to express eYFP without any significant cytotoxic effect, a series of experiments were conducted to assess the optimal output parameters. Post-transduction with eYFP alone, cells were placed into the POLARStar plate reader and the assay performed with fluorescence outputs measured from either top or bottom of the plate. Because no positive controls were applied and p.Phe508del homozygous pAEC have minimal CFTR function, little to no change in fluorescence was expected to occur. Top reading of fluorescence demonstrated spontaneous peaks at the time of iodide injection and that this occurred independent of whether the cells were transduced or not at any MOI. These peaks possibly represented reading artefacts due to the fluid injection scattering light and may act to confound data during analysis (Fig 3A). In contrast, there were fewer instabilities on the bottom reading which provided more stable and consistent data (Fig 3B).

### Discriminating CFTR restoration due to improved trafficking

**Fig 4:**
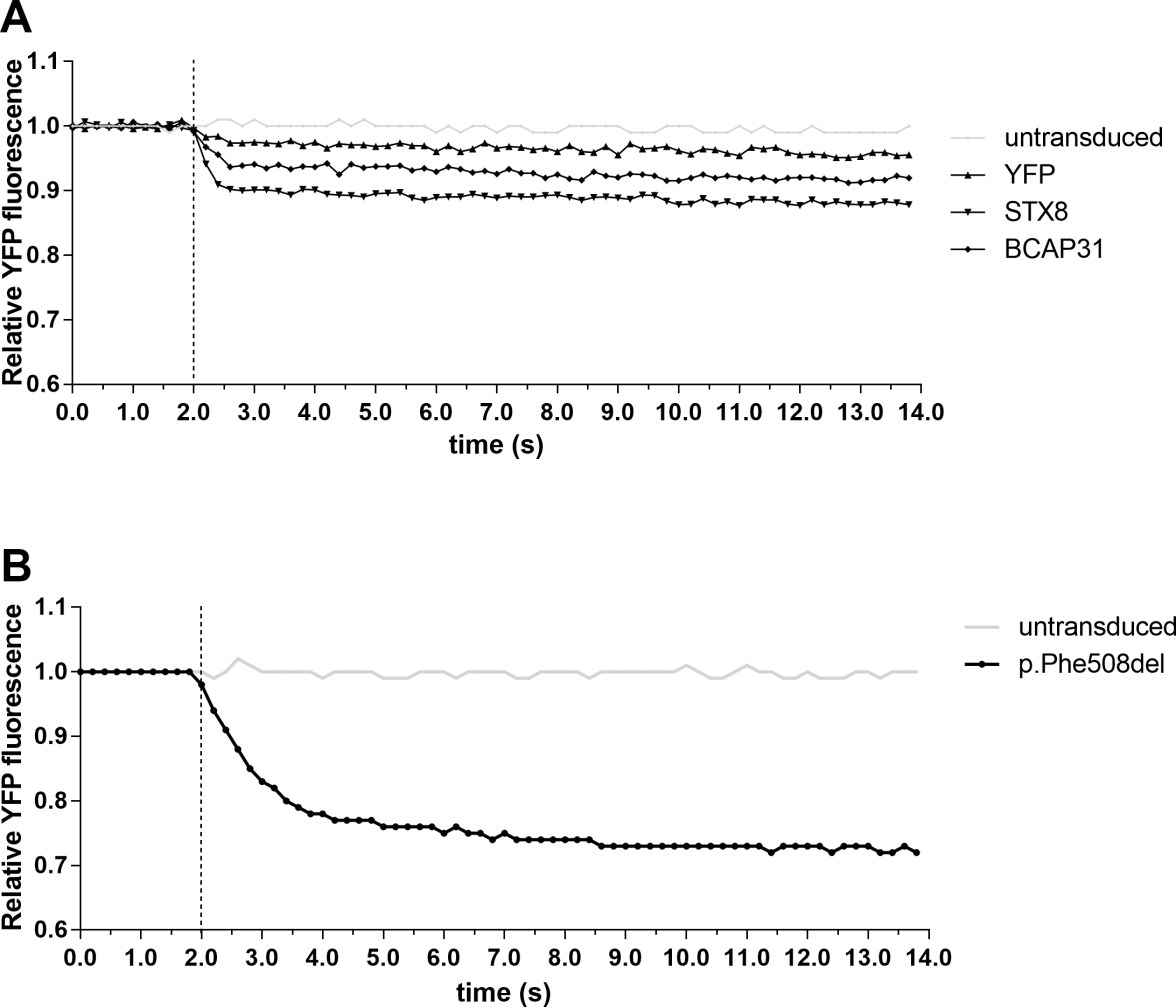
Assessment of adenoviral constructs and CFTR functional restoration using low temperature rescue. **(A)** Primary AEC from children with CF were seeded into 96-well plates, grown to confluence and transduced with eYFP alone or in combination with positive controls STX-8 or BCAP-31 shRNA for 96 hours prior to performing the reporter assay. Knock-down of STX8 or BCAP-31 resulted in functional CFTR indicated by quenching of eYFP fluorescence signals. **(B)** Following transduction of pAEC_CF_ with eYFP and p.Phe508del cells were returned to 37°C incubator for 72 hours, followed by incubation at 27°C for 24 hours prior to halide assay. Halide assay demonstrated activation of the CFTR channels resulting in quenching of the eYFP fluorescence signal. This indicates the feasibility of using halide assay as a tool to measure functional restoration of CFTR channel.

Restoration of p.Phe508del CFTR function can be achieved by overcoming the poor trafficking of the mutated CFTR. Here, the ability of the halide assay to discriminate restoration was first assessed in cells transduced with eYFP and either STX8 or BCAP-31 shRNA to inhibit their production. It was evident that knockdown of either STX8 or BCAP-31 resulted in the rescue of defective CFTR channels in pAEC_CF_ as indicated by the fluorescent quenching (Fig 4A). Therefore, both STX8 and BCAP-31 were used as positive controls in all subsequent experiments.

It has also been previously demonstrated that defective p.Phe508del CFTR channel trafficking can be rescued by culturing cells at lower temperatures [25]. We performed a similar assessment by transducing cells with both eYFP and p.Phe508del cDNA, before incubation at 27°C for 24 hours prior to performing the halide assay. Quenching of the eYFP fluorescent signal was observed (Fig 4B), indicating increased levels of p.Phe508del CFTR were achieved at the cell surface. Overall, data obtained from the transduction with three different positive controls and the recognized temperature rescue method suggest the halide assay is able to discriminate CFTR restoration in pAEC_CF_ homozygous for the p.Phe508del mutation.

### Inter-subject performance of halide reporter assay

**Fig 5:**
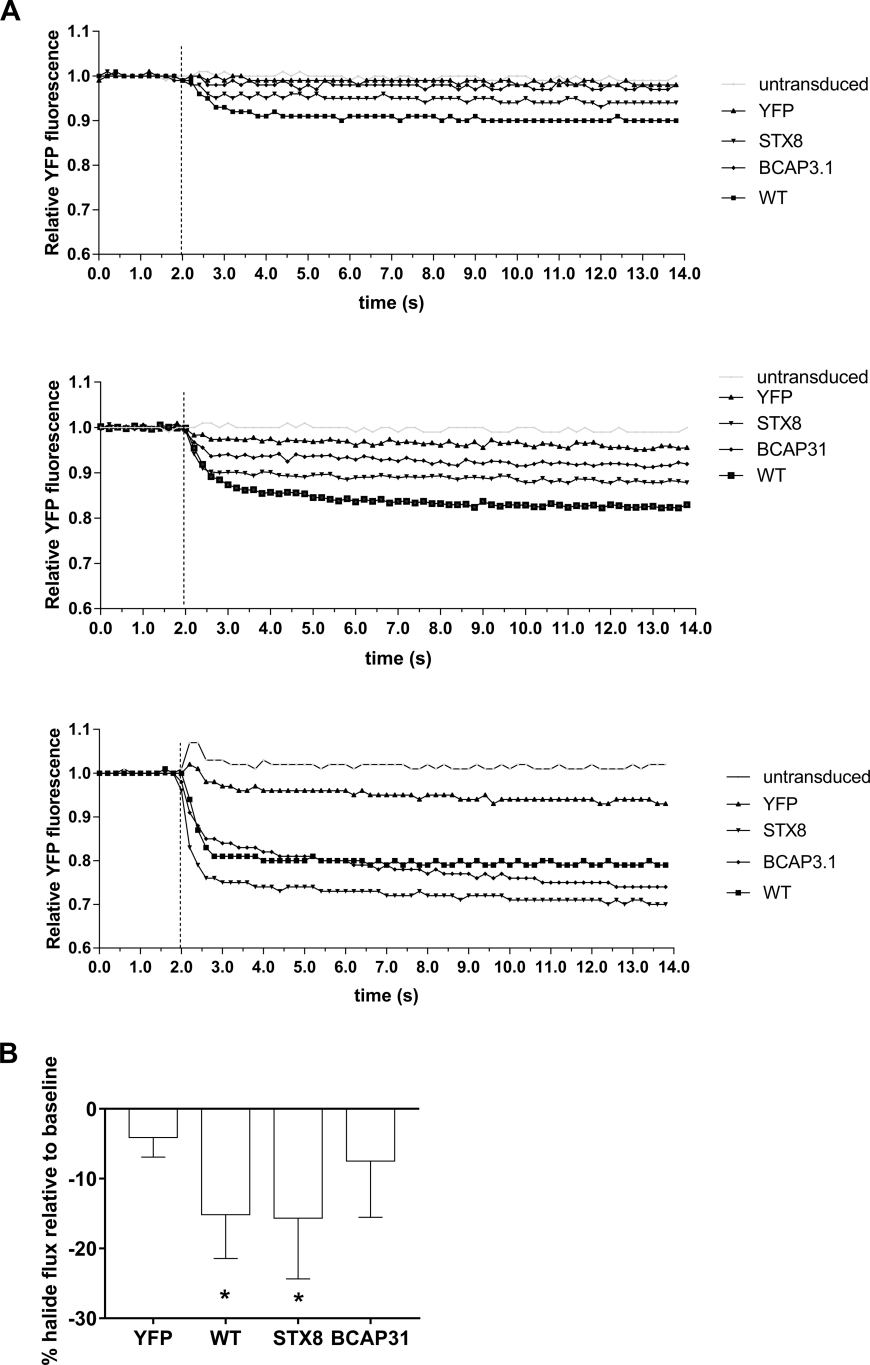
Confirmation of CFTR channel functional restoration in pAEC_CF_. **(A)** A representative of three separate halide assays on pAEC_CF_ cultures illustrating the lowest (top panel), average (middle panel) and highest (bottom panel) level of halide influx. Epithelial cell cultures from a total of 7 children with CF were seeded into 96-well culture plates and transduced with eYFP alone or in combination with STX-8, BCAP-31 shRNA or WT-CFTR for 96 hours. The halide reporter assay was then performed and data analyzed from all 7 cultures to determine the relative quenching of eYFP fluorescence. **(B)** Analysis of data demonstrated that transduction with constructs bearing shRNA for STX-8 or WT-CFTR activated the CFTR channel resulting in subsequent eYFP quenching. This further verified functional restoration of the CFTR channel. However, transduction with construct bearing shRNA for BCAP-31 only resulted in low quenching of eYFP. NOTE: * denotes significant difference at p<0.05.

Once the halide assay was established in our primary cell model, a total of 7 pAEC_CF_ cultures were setup to confirm maximal restoration of CFTR function by transduction with WT-CFTR. Positive controls STX8 and BCAP-31 were also included to assess their capacity for channel restoration compared to WT-CFTR. Fig 5A illustrates representative halide assay readouts from three separate children homozygous for the p.Phe508del mutation. Graphs illustrated the minimum-maximum inter-subject variability in output readings with the lowest (A-top panel), average (middle) and highest level of halide flux (bottom) respectively of the seven subjects assessed. Analysis of pooled data from all subjects demonstrated that transduction with eYFP alone resulted in minimal halide flux (Fig 5B). However, transduction with WT-CFTR or STX8 shRNA resulted in a significant reduction of eYFP fluorescence post-iodide injection, indicating restoration of CFTR function had occurred (p<0.05). Overall, eYFP fluorescence values decreased approximately 15.2 ± 6.29% in response to WT-CFTR transduction, and about 15.7 ± 8.69% post STX8 shRNA. Meanwhile, transduction with BCAP-31 shRNA resulted only in lower quenching of eYFP fluorescence signals (7.49 ± 8.04%).

## Discussion

In this study, we assessed the potential of using a halide reporter assay based around an enhanced eYFP molecule to determine functional CFTR correction in pediatric pAEC from children with CF. Firstly, it was confirmed that pAEC_CF_ could be transduced with high efficiency using an adenoviral construct carrying the eYFP gene, with a positive relationship between transduction efficiency and MOI of the construct used. Our findings also suggested minimal cytotoxicity in pAEC_CF_ following transduction with eYFP alone or in combination with WT-CFTR or STX8, with BCAP-31 shRNA appearing to induce some level of cellular stress. Evaluation of different approaches to assay readouts verified that measuring eYFP fluorescence from the bottom of the microplate resulted in minimal signal instabilities. In contrast, reading the plate from the top resulted in spontaneous peaks at the time of iodide injection, independent of any adenoviral construct being added, which would confound data during analysis.

Minimal or no reduction in eYFP fluorescence readouts occurred for pAEC_CF_ that were transduced with eYFP only. Whereas, transduction with eYFP in combination with WT-CFTR or either of the positive controls did result in fluorescence quenching, indicating increased CFTR function had been achieved and these were corroborated by separate assays performed after low temperature rescue of p.Phe508del CFTR channel trafficking. Inter-subject variability in output readings are most likely reflective of the inherent phenotypic variance in human CF populations, even within homozygous mutation genotypes. This further highlights the need for individual-based analysis of any potential therapy and is the subject of a study by our group with particular implications for personalized high-throughput screening of CFTR therapeutics across all phenotypic classes of CFTR function.

The gold standard method of measuring ion transport via Ussing chamber provides a more accurate functional assessment of the CFTR channel. However, this method requires cells grown at air-liquid interface, which can often take up to 28 days before any assessment is performed, thus limiting the feasibility of this method as a high-throughput screen. In contrast, the halide assay can be adapted into various small-scale, high-throughput platforms using either 96- or 384-well plate formats for the rapid assessment of CFTR functionality. Miniaturization combined with the use of patient derived AEC, which provides reflection on the phenotypic variance within the CF population, can then be adapted for a more personalized approach for CF therapy to cater especially for individuals with rare CFTR mutations. However, the main limitation to this method is in using a submerged culture format, which is often not reflective of the differentiated airways *in vivo*. Nonetheless, the halide assay is highly adaptable and ideal for the rapid screening of therapeutic compounds targeting specific CFTR mutations. Results generated by this assay can be subsequently verified using Ussing to corroborate activity in a differentiated airway setting.

This study explored an alternative halide assay first developed by Verkman [7], where a halide ion was used to quench a reporter gene expression that is co-expressed with the WT-CFTR gene. Most high throughput screening studies using fluorescent techniques to evaluate compounds for CFTR corrective capabilities have utilized Swiss 3T3 mouse fibroblasts [26,27] or Fischer rat thyroid cells [22,28,29] that were then stably transfected to express p.Phe508del. Other studies have either used human bronchial cell lines such as CFBE41o- [29] or cultures of human bronchial epithelial cells established from lung resections or transplant tissues for confirmation studies of CFTR expression and function [27,30]. However, the use of immortalized cell lines including those that were virally transformed may not always reflect characteristics of primary airway epithelial cells and individual patient responses to different therapeutic compounds cannot be captured when generic cell lines or animal cells are used as part of a HTS platform. Recent studies on CFTR rescue have been performed using AECs isolated from bronchial tissues following biopsy or lung transplantation in adults [31–34]. However, it is known that the lung environment and subsequent disease pathogenesis greatly differ between children and adults and there remains paucity of data on the assessment of CFTR channel functionality using AECs from pediatric populations. Significantly, this study using pAEC_CF_ overcomes these limitations and thus advances the field of CFTR assessment by making personalized high-throughput screening a reality. Furthermore, the use of cell cultures from a pediatric population, as in this study, illustrates the potential of this screening platform in early life interventional therapy.

There have been significant advances in the development of small molecules to target the defective CFTR at molecular level with promising results [35]. Two of these molecules, the CFTR potentiator ivacaftor and the CFTR corrector lumacaftor have been recently approved for therapeutic use in several countries. However, due to the many mutations of the CFTR gene, there are ongoing investigations for additional CFTR modulators to correct rarer or functionally unique CFTR mutations. These investigations would be significantly benefitted by a high-throughput screening platform that could assess CFTR functional restoration in a scale that uses fewer cells and over a shorter period.

Other methods that have been used to measure CFTR function *in vitro* include the fluorescence-resonance energy transfer (FRET)-based assay [31] and fluorescent membrane potential probe in FlexStation-based membrane potential assay [36,37]. Despite the widespread use of FRET-based assay technology for high-throughput ion channel drug discovery, there remains significant limitations including low signal to noise ratio as well specific requirements pertaining to the physical properties of the fluorescence labels in order for the assay’s accuracy [38]. Encouragingly, with improved technologies and new innovations, these limitations are progressively being overcome. The fluorescent membrane potential probe assay provides further advancement in the monitoring of CFTR function with it ease of use. However, the assay was initially optimized in cell lines manipulated to highly express CFTR and has only very recently been translated to patient derived samples. Further validation will be required, specifically in a very young pediatric cohort.

Here, we have provided proof of concept of a small-scale halide assay using primary pediatric airway cultures for assessing CFTR restoration. This small scale assay could be further adapted for a high-throughput screening format to allow for personalized (individual patient-specific) assessment of CFTR correction using an existing and/or novel compound(s). This assay can then be setup either for personalized screening of multiple compounds or for testing a specific compound for a range of CFTR mutations. Moreover, any prospective therapy can be assessed in adult-derived airway epithelial cells to determine its potential benefit over age and disease progression.

The benefits of using halide-sensitive fluorescent reporter include capacity to miniaturize the format to 384-well, the ability to measure heterogeneous CFTR combinations and an apparent high sensitivity of detection for functional CFTR expression [8]. Notwithstanding the benefits of using pediatric primary cultures, these cultures also have their limitations. Cells could be only be passaged up to 5 times before reaching terminal expansion potential under traditional culturing approaches [19]. A new method using of Rho-associated protein kinase (ROCK) inhibitor in combination with mitotically-inactive fibroblast feeder layers could be adapted to overcome this challenge [39]. A recent study demonstrated that applying such a combination in endotracheal-derived cells increased their growth rate and also produced functional airway epithelial cells for various applications [40]. Despite the potential of this method, it remains relatively new and we are currently validating the halide assay under these culture conditions for comparison to unmodified pAEC_CF_ cultures.

Recently, other studies have used methods including three-dimensional organoids and induced pluripotent stem cells (iPSC) to assess CFTR function in patient-specific settings [17,40–44]. The study by Dekkers *et al*. used an intestinal organoid system to study CFTR function in such a personalized setting [17]. In healthy-derived organoids, application of the CFTR stimulant forskolin caused swelling of the sphere’s size, reflecting normal CFTR function, whilst the same degree of swelling was not observed in CF-derived organoids. Organoids were also previously used by Schwank *et al*. to study functional repair of CFTR by CRISPR/Cas9 editing system [41]. They observed swelling in the corrected organoids following administration of forskolin, whereas no swelling was detected in control uncorrected organoids. Despite promising findings, differences between the low turnover of airway cells compared to the intestine, the low CFTR expression that might affect the rate of forskolin-induced swelling, and the possible presence of compensatory non-CFTR channels [45] all raises queries to their appropriateness as an airway surrogate. Excitingly, recent work by two groups have reported the successful formation of airway organoids and their potential application for HTS [46,47]. Even with these advancements, organoid modelling remains relatively new and additional research on its relevance to assess CFTR function that reflect/mimic *in vivo* conditions is required.

Another field that has gained momentum in recent years is the use of iPSC-derived cell cultures as they can be differentiated into a variety of tissue-specific cell types including airway epithelium [42–44]. Firth *et al*. successfully demonstrated differentiation of human iPSC into polarized airway epithelium with ciliated cells and functional CFTR activity [42]. As iPSCs can be generated from a blood sample in patient-specific settings, this model has attracted a lot of interest as an unlimited source of patient-specific airway epithelial cells. Despite the potential, induction of iPSC into airway epithelium is typically laborious and involves multiple, meticulous steps to ensure the production of appropriate cues that allow cells to follow desired lineages. Challenges regarding the need for homogeneous cell population with no contamination by undifferentiated cells and the cost and complexity of the technique all suggest that this model system still requires considerable refinement.

## Conclusions

This study has demonstrated that the eYFP-halide reporter assay is an efficient technique to evaluate successfully restored and activated p.Phe508del in CF primary airway epithelial cells. More importantly, due to its small-scaled format, the use of patient-specific primary airway cells and the reporter readout, this assay may have potential as a personalized HTS tool to screen and identify the most optimal therapy for particular individuals with CF are amenable to, irrespective of the mutation causing the disease.
